# Crystal structure of bis­[octa­kis­(di­methyl sulfoxide-κ*O*)­ytterbium(III)] penta­bromido­plumbate(II) tribromide di­methyl sulfoxide monosolvate: a ytterbium-doped lead halide perovskite precursor

**DOI:** 10.1107/S2056989023002852

**Published:** 2023-03-31

**Authors:** Takumi Kinoshita, Kanna Fukumoto, Hiroshi Segawa

**Affiliations:** a Graduate School of Arts and Sciences, The University of Tokyo, 3-8-1 Komaba, Meguro-ku, Tokyo, 153-8902, Japan; b Research Center for Advanced Science and Technology (RCAST), The University of, Tokyo 4-6-1, Komaba, Meguro-ku, Tokyo, 153-8904, Japan; University of Neuchâtel, Switzerland

**Keywords:** lead halide perovskite, ytterbium, photoluminescence, crystal structure

## Abstract

A mixture of PbBr_2_ and YbBr_3_·*n*H_2_O in a dimethyl sulfoxide (DMSO) solution yielded single crystals of a lead halide perovskite precursor with ytterbium, bis­[octa­kis­(di­methyl sulfoxide)­ytterbium(III)]penta­bromido­plumbate(II) tri­bromide with di­methyl sulfoxide as co-crystallite, [Yb(C_2_H_6_OS)_8_][PbBr_5_]_0.5_Br_1.5_·0.5C_2_H_6_OS. The complex ions PbBr_5_^3−^ and Yb(DMSO)_8_^3+^ are present in the crystal together with three Br^−^ ions and DMSO mol­ecules. X-ray crystallography revealed that the Br^−^ ions in YbBr_3_ are replaced by the solvent and bound to a Pb^II^ atom or remain free. The presence of PbBr_5_^3−^ units, which are mol­ecular ions with a square-pyramidal structure, is also observed. These single crystals react with a caesium chloride solution, exhibiting near-infrared (NIR) luminescence by visible photoexcitation, suggesting the formation of Yb^3+^-doped lead halide perovskites (CsPbBr_3-*x*_Cl_*x*_·Yb^3+^).

## Abstract 2

A mixture of PbBr_2_ and YbBr_3_·*n*H_2_O in a dimethyl sulfoxide (DMSO) solution yielded single crystals of a lead halide perovskite precursor with ytterbium, bis­[octa­kis­(di­methyl sulfoxide)­ytterbium(III)]penta­bromido­plumbate(II) tribromide with di­methyl sulfoxide as co-crystallite. These single crystals react with a caesium chloride solution, exhibiting near-infrared (NIR) luminescence by visible photoexcitation, suggesting the formation of Yb^3+^-doped lead halide perovskites

## 1. Chemical context

Lead halide perovskite crystals have attracted considerable attention in the fields of solar cells and optoelectronics (Lee *et al.*, 2012 ▸; Burschka *et al.*, 2013 ▸; Fu *et al.*, 2019 ▸). Lead halide perovskite crystals have been investigated extensively owing to their facile solution-phase fabrication, high energy-conversion efficiency, and characteristic photoresponse. Lead halide perovskites can easily be prepared by spin coating microcrystalline thin films in solution. 
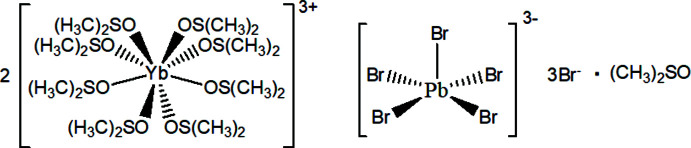


Highly polar solvents, such as di­methyl­formamide (DMF) and dimethyl sulfoxide (DMSO), are used in fabricating perovskite thin films by solution processing. Typically, these solvents are removed by thermal annealing using a hot plate or air drying after spin coating, and crystal growth proceeds as the solvent becomes supersaturated. The crystal morphology and crystalline phase depend on the annealing temperature and treatment time (Tenailleau *et al.*, 2019 ▸; Bi *et al.*, 2014 ▸; Xiao *et al.*, 2014 ▸; Jung *et al.*, 2019 ▸). The morphology of perovskite films, such as the film thickness and grain boundaries, significantly affects the performance of solar cells. Complex formation between Pb atoms and solvent mol­ecules in the perovskite precursor solution significantly influences the film morphology (Ozaki *et al.*, 2017 ▸; Wakamiya *et al.*, 2014 ▸; Ozaki *et al.*, 2019 ▸). The addition of CH_3_NH_3_I dissolved in 2-propanol to 1D crystals displaced the DMF solvent, forming a 3D perovskite structure. The addition of CH_3_NH_3_I dissolved in 2-propanol to these 1D crystals suspended in DMF solvent forms a 3D perovskite structure (Wakamiya *et al.*, 2014 ▸). The CH_3_NH_3_I-PbI_2_-DMF inter­mediate formed by CH_3_NH_3_I addition was also observed during thermal annealing. DMF coordination with the inter­mediate is thought to be responsible for Ostwald ripening (Guo *et al.*, 2016 ▸). Additionally, when DMSO was used as the solvent, a PbI_2_-(DMSO)_2_ complex was formed, in which DMSO was more strongly coordinated to PbI_2_ than DMF (Miyamae *et al.*, 1980 ▸).

Lead halide perovskite thin films have been investigated extensively for solar cells and various other fields, including optoelectronics. Recently, the efficient luminescence of rare-earth elements using a lead halide perovskite as an optical absorption antenna was reported by doping ytterbium into a 3D CsPbBr_*x*_Cl_3-*x*_ perovskite (Kroupa *et al.*, 2018 ▸; Erickson *et al.*, 2019 ▸). However, the crystal structure of lead halide perovskites doped with rare-earth elements and their mechanism of formation remains unclear. In this study, precursor single-crystals of a lead halide perovskite doped with rare-earth elements, bis­[octa­kis­(di­methyl sulfoxide)­ytterbium(III)] penta­bromido­plumbate(II) tribromide di­methyl sulfoxide solvate, were successfully prepared, and the structure of the precursor crystal was determined.

## 2. Structural commentary

**Figure 1:**
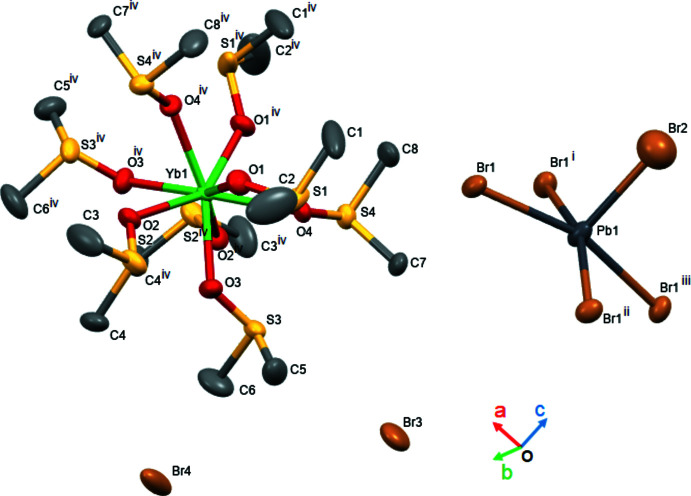
Arrangement of [Yb(DMSO)_8_]^3+^, [PbBr_3_]^2−^ and free Br^−^ anions in the precursor crystal, with displacement ellipsoids at the 50% probability level. The disordered DMSO mol­ecule is omitted for clarity. Symmetry codes: (i) *y*, 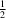 − *x*, *z*; (ii) 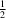 − *y*, *x*, *z*; (iii) 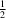 − *x*, 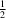 − *y*, *z*; (iv) 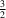 − *x*, 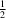 − *y*, *z*.

The obtained structure exhibits an alternating sequence of PbBr_5_^3−^ and 2[Yb(DMSO)_8_]^3+^ units (Figs. 1 ▸ and 2 ▸). The [Yb(DMSO)_8_]^3+^ unit is considered to possess three Br^−^ (Br3, Br4) ions as counter-anions. Inter­estingly, the PbBr_5_^3−^ unit exhibits a square-pyramidal structure. Lead halide compounds often show lead-centered octa­hedral structures, and there have been no previous reports of the PbBr_5_^3−^ mol­ecular ion with a square-pyramidal geometry. The free atom Br3 is located on the straight line of the Br2—Pb1 bond, and the Pb1⋯Br3 distance is 6.781 (9) Å (Fig. 1 ▸). The free Br3 atom is located at a distance more than twice that of Br2 in the Pb1—Br2 bond [2.814 (4) Å], suggesting that there is no Pb1—Br3 inter­action.

**Figure 3:**
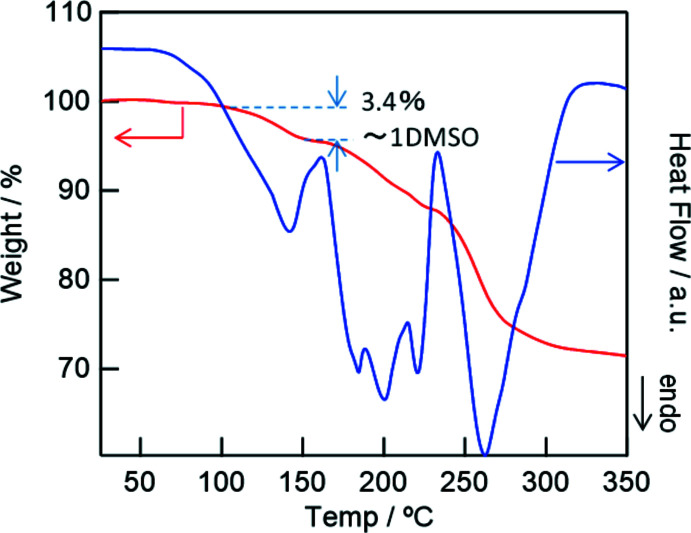
TG–DTA curves acquired under a nitro­gen atmosphere.

The DMSO mol­ecule as co-crystallite is disordered, and the exact configuration was difficult to determine. Thermogravimetric analysis (TG–DTA) of the crystals revealed a weight loss of 3.4% at approximately 410 K, with an endothermic peak, corresponding to a dissociation of 0.5 equivalents of DMSO relative to Yb (theoretical value 3.1 wt%) (Fig. 3 ▸). The crystal structure resembles that of a 1D perovskite with a series of (Pb*X*_5_^3−^) units (Wang *et al.*, 1995 ▸). However, the weak inter­actions between the Br^−^ ions and DMSO mol­ecules in the gaps between the (Pb*X*_5_^3−^) units prevents the 1D perovskite from bridging. All halogen ions were lost when YbBr_3_ was added, and DMSO is coordinated to the Yb^III^ atom instead. Several Br^−^ ions react with PbBr_2_ to form PbBr_5_^3−^, and therefore YbBr_3_ has served as a source of halogen ions in the lead halide perovskite framework.

## 3. Photophysical analysis

**Figure 4:**
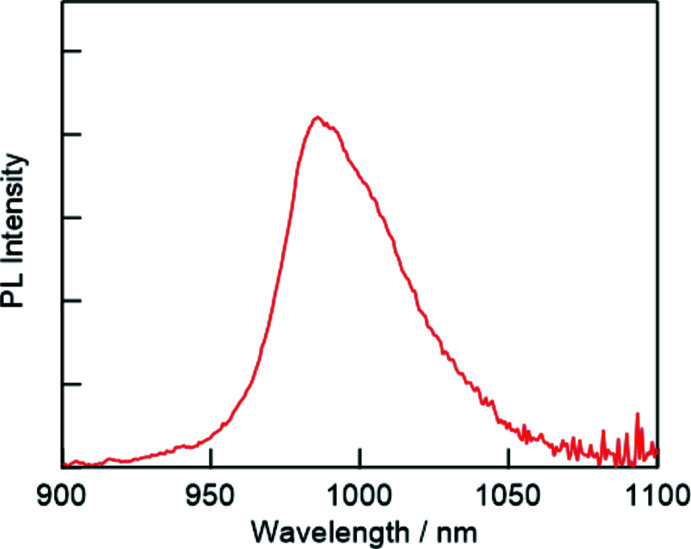
Near-infrared emission from Yb^3+^ after treatment of the precursor crystals with a CsCl methanol solution.

The precursor crystal did not exhibit any luminescence upon irradiation with visible light. In contrast, the dropwise addition of a methanol solution containing caesium chloride to the precursor crystals, followed by annealing at 473 K for 5 min, resulted in the formation of light-yellow microcrystals. The microcrystals exhibited Yb^3+^-derived near-infrared (NIR) emission at 980 nm upon photoexcitation at 400 nm (Fig. 4 ▸). This indicates that the precursor crystals reacted with caesium chloride, and Yb^3+^-doped 3D lead halide perovskite crystals (CsPbBr_3-x_Cl_x_·Yb^3+^) (Erickson *et al.*, 2019 ▸) were formed. The NIR luminescence of doped Yb^3+^ was observed, in addition to the visible-light absorption of the lead halide perovskite crystals.

## 4. Database survey

The Inorganic Crystal Structure Database (ICSD) (ICSD, 2023 ▸) did not include any closely related structures. For [Yb(DMSO)_8_]^3+^ units, tetra­kis­[1,4-bis­(phenyl­sulfin­yl)butane]­ytterbium(III) triperchlorate (Li *et al.*, 2004 ▸) and *catena*-[octa­kis­(di­methyl sulfoxide)­ytterbium hepta­kis­(di­methyl sulfoxide)­ytterbium hexa­kis­(μ_3_-sulfido)­dodeca­kis­(μ_2_-sulfido)­hexa­sulfido­hexa­silverhexa­tungsten] (Zhang *et al.*, 2011 ▸) are present in the database.

## 5. Synthesis and crystallization

**Figure 5:**
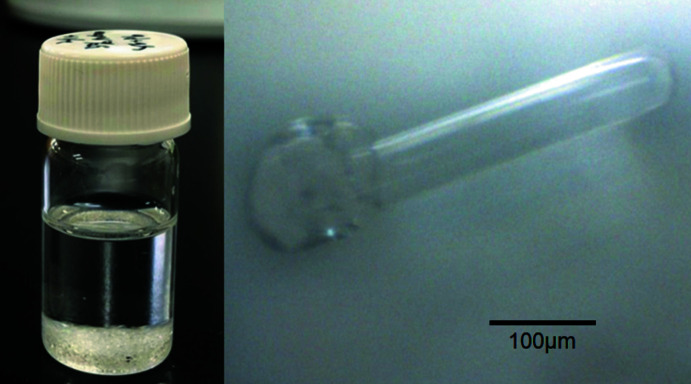
Photographs of crystals in solution and of the single crystal used for the measurement.

PbBr_2_ and YbBr_3_·*n*H_2_O were dissolved in DMSO (anhydrous, Fujifilm Wako Pure Chemicals) to prepare a 0.5 *M* solution. The solution was heated to 373 K using a hot plate; acetone was added gradually to obtain colourless needle-like crystals (Fig. 5 ▸).

## 6. Refinement

The crystal data, data collection, and structural refinement details are summarized in Table 1 ▸. Because the precursor crystals contain numerous heavy atoms, it was difficult to analyze the residual electrons of these atoms; therefore, an empirical absorption correction using spherical harmonics was applied. The residual electron densities *Δρ*_max_ and *Δρ*_min_ of 8.97 and −1.78 e Å^−3^ are located 0.912 and 0.918 Å, respectively, from the Pd atom. H atoms were positioned geom­etrically (C—H = 0.98 Å) and refined as riding with *U*_iso_(H) = 1.5*U*_eq_(C). Various atoms were refined with fixed occupancies: S5 (0.25) C9 (0.5) H9*A* (0.5) H9*B* (0.5) H9*C* (0.5).

## Supplementary Material

Crystal structure: contains datablock(s) I. DOI: 10.1107/S2056989023002852/tx2065sup1.cif

Structure factors: contains datablock(s) I. DOI: 10.1107/S2056989023002852/tx2065Isup2.hkl

CCDC reference: 2251523

Additional supporting information:  crystallographic information; 3D view; checkCIF report

## Figures and Tables

**Figure 2:**
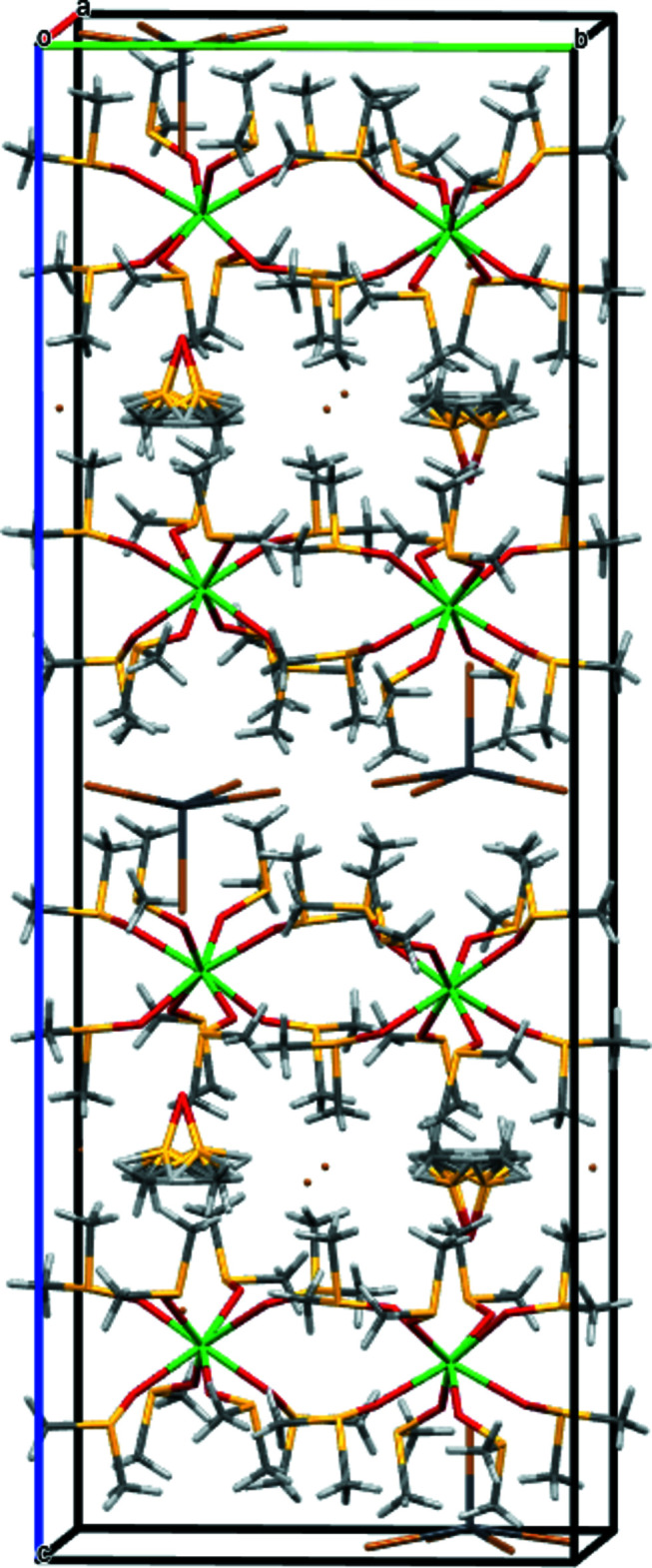
Perspective view of the precursor crystal structure along [100].

**Table 1:** Experimental details

Crystal data	
Chemical formula	[Yb(C_2_H_6_OS)_8_][PbBr_5_]_0.5_Br_1.5_·0.5C_2_H_6_OS
*M*_r_	1260.36
Crystal system, space group	Tetragonal, *P*4/*n* *c* *c*
Temperature (K)	93
*a*, *c* (Å)	14.3940 (2), 40.6538 (8)
*V* (Å^3^)	8422.9 (3)
*Z*	8
Radiation type	Cu *K*α
μ (mm^−1^)	16.57
Crystal size (mm)	0.50 × 0.36 × 0.04

Data collection	
Diffractometer	XtaLAB Synergy, Dualflex, HyPix
Absorption correction	Multi-scan (*CrysAlis PRO*; Rigaku OD, 2019 ▸)
*T*_min_, *T*_max_	0.195, 1.000
No. of measured, independent and observed [*I* > 2σ(*I*)] reflections	23286, 4274, 3579
*R*_int_	0.108
(sin θ/λ)_max_ (Å^−1^)	0.626

Refinement	
*R*[*F* ^2^ > 2σ(*F* ^2^)], *wR*(*F* ^2^), *S*	0.080, 0.225, 1.08
No. of reflections	4274
No. of parameters	203
H-atom treatment	H-atom parameters constrained
Δρ_max_, Δρ_min_ (e Å^−3^)	8.97, −1.78

Computer programs: *CrysAlis PRO* (Rigaku OD, 2019 ▸), *OLEX2.solve* (Bourhis *et al.*, 2015 ▸), *SHELXL2018/3* (Sheldrick, 2015 ▸) and *OLEX2* (Dolomanov *et al.*, 2009 ▸).

## supplementary crystallographic information

### Crystal data

**Table:**

[Yb(C_2_H_6_OS)_8_][PbBr_5_]_0.5_Br_1.5_·0.5C_2_H_6_OS	*D*_x_ = 1.988 Mg m^−^^3^
*M**_r_* = 1260.36	Cu *K*α radiation, λ = 1.54184 Å
Tetragonal, *P*4/*n**c**c*	Cell parameters from 8591 reflections
*a* = 14.3940 (2) Å	θ = 4.4–74.7°
*c* = 40.6538 (8) Å	µ = 16.57 mm^−^^1^
*V* = 8422.9 (3) Å^3^	*T* = 93 K
*Z* = 8	Plate, colourless
*F*(000) = 4864	0.50 × 0.36 × 0.04 mm

### Data collection

**Table:**

XtaLAB Synergy, Dualflex, HyPix diffractometer	3579 reflections with *I* > 2σ(*I*)
Detector resolution: 10.0000 pixels mm^-1^	*R*_int_ = 0.108
ω scans	θ_max_ = 74.9°, θ_min_ = 2.2°
Absorption correction: multi-scan (CrysAlisPro; Rigaku OD, 2019)	*h* = −16→17
*T*_min_ = 0.195, *T*_max_ = 1.000	*k* = −12→17
23286 measured reflections	*l* = −35→50
4274 independent reflections	

### Refinement

**Table:**

Refinement on *F*^2^	0 restraints
Least-squares matrix: full	Hydrogen site location: inferred from neighbouring sites
*R*[*F*^2^ > 2σ(*F*^2^)] = 0.080	H-atom parameters constrained
*wR*(*F*^2^) = 0.225	*w* = 1/[σ^2^(*F*_o_^2^) + (0.152*P*)^2^ + 25.6573*P*] where *P* = (*F*_o_^2^ + 2*F*_c_^2^)/3
*S* = 1.08	(Δ/σ)_max_ = 0.001
4274 reflections	Δρ_max_ = 8.97 e Å^−^^3^
203 parameters	Δρ_min_ = −1.78 e Å^−^^3^

### Special details

**Table:**

Geometry. All esds (except the esd in the dihedral angle between two l.s. planes) are estimated using the full covariance matrix. The cell esds are taken into account individually in the estimation of esds in distances, angles and torsion angles; correlations between esds in cell parameters are only used when they are defined by crystal symmetry. An approximate (isotropic) treatment of cell esds is used for estimating esds involving l.s. planes.

### Fractional atomic coordinates and isotropic or equivalent isotropic displacement parameters (Å^2^)

**Table:**

	*x*	*y*	*z*	*U*_iso_*/*U*_eq_	Occ. (<1)
Pb1	0.250000	0.250000	0.50704 (2)	0.0340 (3)	
Yb1	0.750000	0.250000	0.37362 (2)	0.0190 (3)	
Br1	0.43356 (6)	0.34068 (6)	0.49553 (2)	0.0349 (3)	
Br3	0.250000	0.250000	0.34023 (5)	0.0559 (6)	
Br4	0.49813 (6)	0.50187 (6)	0.250000	0.0608 (7)	
S4	0.54958 (13)	0.14251 (13)	0.40874 (5)	0.0303 (4)	
S3	0.53965 (14)	0.27929 (16)	0.33522 (5)	0.0345 (5)	
S2	0.76666 (17)	0.46366 (15)	0.33661 (5)	0.0377 (5)	
S1	0.64007 (15)	0.41439 (19)	0.42370 (6)	0.0500 (7)	
O3	0.6392 (4)	0.3125 (4)	0.33952 (15)	0.0344 (12)	
O1	0.7248 (4)	0.3778 (4)	0.40597 (15)	0.0356 (12)	
O4	0.6108 (4)	0.2258 (4)	0.40211 (14)	0.0284 (11)	
O2	0.8176 (4)	0.3738 (4)	0.34489 (14)	0.0314 (12)	
C7	0.4353 (5)	0.1886 (7)	0.4131 (2)	0.0363 (18)	
H7A	0.435947	0.238849	0.429408	0.055*	
H7B	0.393082	0.139411	0.420479	0.055*	
H7C	0.414022	0.212997	0.391917	0.055*	
C4	0.7484 (6)	0.4587 (9)	0.2931 (3)	0.048 (3)	
H4A	0.807678	0.446497	0.281992	0.071*	
H4B	0.723157	0.518084	0.285400	0.071*	
H4C	0.704475	0.408721	0.287950	0.071*	
O5	0.750000	0.750000	0.3018 (5)	0.100 (9)	
C8	0.5663 (7)	0.1162 (8)	0.4512 (3)	0.050 (2)	
H8A	0.628770	0.090851	0.454460	0.075*	
H8B	0.520014	0.070349	0.458222	0.075*	
H8C	0.559160	0.173003	0.464263	0.075*	
C5	0.4723 (7)	0.3819 (8)	0.3353 (3)	0.052 (3)	
H5A	0.476672	0.411699	0.356951	0.078*	
H5B	0.407268	0.366359	0.330720	0.078*	
H5C	0.495481	0.424428	0.318416	0.078*	
C6	0.5276 (11)	0.2517 (8)	0.2925 (3)	0.060 (3)	
H6A	0.532561	0.308699	0.279429	0.090*	
H6B	0.466864	0.222907	0.288694	0.090*	
H6C	0.576829	0.208406	0.285983	0.090*	
C3	0.8528 (10)	0.5533 (7)	0.3366 (3)	0.065 (4)	
H3A	0.888786	0.550183	0.357047	0.097*	
H3B	0.822330	0.614034	0.335028	0.097*	
H3C	0.894404	0.544800	0.317779	0.097*	
C1	0.6579 (9)	0.3911 (13)	0.4651 (3)	0.080 (5)	
H1A	0.659188	0.323712	0.468536	0.119*	
H1B	0.607311	0.418331	0.478018	0.119*	
H1C	0.717213	0.418025	0.472066	0.119*	
C2	0.6643 (11)	0.5360 (10)	0.4257 (4)	0.095 (6)	
H2A	0.731670	0.545755	0.425340	0.143*	
H2B	0.638504	0.561609	0.446075	0.143*	
H2C	0.636020	0.567247	0.406771	0.143*	
S5	0.7808 (8)	0.7165 (8)	0.2655 (2)	0.0300 (17)	0.25
C9	0.684 (2)	0.686 (2)	0.2475 (5)	0.075 (11)	0.5
H9A	0.697334	0.666771	0.224902	0.113*	0.5
H9B	0.641419	0.739136	0.247289	0.113*	0.5
H9C	0.655988	0.634559	0.259618	0.113*	0.5
Br2	0.250000	0.250000	0.57625 (11)	0.0958 (12)	

### Atomic displacement parameters (Å^2^)

**Table:**

	*U*^11^	*U*^22^	*U*^33^	*U*^12^	*U*^13^	*U*^23^
Pb1	0.0241 (3)	0.0241 (3)	0.0539 (5)	0.000	0.000	0.000
Yb1	0.0228 (3)	0.0217 (3)	0.0124 (4)	−0.00053 (15)	0.000	0.000
Br1	0.0260 (4)	0.0325 (5)	0.0460 (5)	−0.0026 (3)	−0.0052 (3)	0.0051 (3)
Br3	0.0723 (10)	0.0723 (10)	0.0233 (10)	0.000	0.000	0.000
Br4	0.0775 (10)	0.0775 (10)	0.0274 (8)	0.0460 (12)	0.0088 (6)	0.0088 (6)
S4	0.0305 (9)	0.0285 (8)	0.0320 (10)	−0.0019 (7)	0.0069 (7)	0.0006 (7)
S3	0.0304 (9)	0.0498 (11)	0.0235 (9)	−0.0071 (8)	−0.0053 (7)	0.0102 (9)
S2	0.0557 (12)	0.0316 (10)	0.0257 (10)	−0.0024 (8)	−0.0002 (9)	0.0059 (8)
S1	0.0289 (10)	0.0675 (15)	0.0536 (14)	0.0111 (9)	−0.0110 (9)	−0.0388 (12)
O3	0.027 (3)	0.043 (3)	0.033 (3)	−0.001 (2)	−0.006 (2)	0.010 (2)
O1	0.042 (3)	0.037 (3)	0.028 (3)	0.004 (3)	0.002 (3)	−0.010 (2)
O4	0.026 (3)	0.033 (3)	0.025 (3)	−0.006 (2)	0.003 (2)	0.008 (2)
O2	0.037 (3)	0.031 (3)	0.026 (3)	−0.001 (2)	−0.001 (2)	0.010 (2)
C7	0.021 (3)	0.050 (5)	0.038 (4)	−0.002 (3)	0.004 (3)	0.009 (4)
C4	0.044 (5)	0.065 (7)	0.035 (5)	0.001 (4)	−0.013 (4)	0.016 (5)
O5	0.137 (14)	0.137 (14)	0.026 (9)	0.000	0.000	0.000
C8	0.039 (5)	0.069 (6)	0.041 (5)	−0.005 (4)	0.005 (4)	0.027 (5)
C5	0.041 (5)	0.068 (7)	0.048 (6)	0.020 (5)	0.010 (4)	0.011 (5)
C6	0.088 (9)	0.063 (7)	0.030 (5)	−0.009 (5)	−0.015 (6)	−0.008 (4)
C3	0.099 (9)	0.042 (5)	0.053 (6)	−0.036 (6)	−0.018 (6)	0.013 (5)
C1	0.067 (7)	0.139 (13)	0.033 (5)	−0.054 (8)	0.013 (5)	−0.018 (7)
C2	0.088 (10)	0.074 (9)	0.124 (13)	0.039 (8)	−0.033 (9)	−0.066 (9)
S5	0.030 (7)	0.026 (7)	0.034 (4)	0.008 (2)	−0.006 (3)	0.005 (3)
C9	0.10 (2)	0.11 (2)	0.018 (9)	−0.06 (2)	0.002 (10)	−0.030 (11)
Br2	0.1033 (18)	0.1033 (18)	0.081 (3)	0.000	0.000	0.000

### Geometric parameters (Å, º)

**Table:**

Pb1—Br1	2.9839 (9)	C7—H7A	0.9800
Pb1—Br1^i^	2.9840 (9)	C7—H7B	0.9800
Pb1—Br1^ii^	2.9840 (9)	C7—H7C	0.9800
Pb1—Br1^iii^	2.9840 (9)	C4—H4A	0.9800
Pb1—Br2	2.814 (4)	C4—H4B	0.9800
Yb1—S3^iv^	3.4324 (19)	C4—H4C	0.9800
Yb1—S3	3.4325 (19)	O5—S5	1.616 (18)
Yb1—S2^iv^	3.432 (2)	C8—H8A	0.9800
Yb1—S2	3.432 (2)	C8—H8B	0.9800
Yb1—O3	2.297 (5)	C8—H8C	0.9800
Yb1—O3^iv^	2.297 (5)	C5—H5A	0.9800
Yb1—O1	2.290 (6)	C5—H5B	0.9800
Yb1—O1^iv^	2.290 (6)	C5—H5C	0.9800
Yb1—O4	2.341 (5)	C6—H6A	0.9800
Yb1—O4^iv^	2.341 (5)	C6—H6B	0.9800
Yb1—O2^iv^	2.342 (5)	C6—H6C	0.9800
Yb1—O2	2.342 (5)	C3—H3A	0.9800
S4—O4	1.512 (6)	C3—H3B	0.9800
S4—C7	1.783 (8)	C3—H3C	0.9800
S4—C8	1.784 (10)	C1—H1A	0.9800
S3—O3	1.521 (6)	C1—H1B	0.9800
S3—C5	1.767 (10)	C1—H1C	0.9800
S3—C6	1.790 (11)	C2—H2A	0.9800
S2—O2	1.525 (6)	C2—H2B	0.9800
S2—C4	1.792 (11)	C2—H2C	0.9800
S2—C3	1.789 (10)	S5—C9	1.63 (3)
S1—O1	1.511 (6)	C9—H9A	0.9800
S1—C1	1.734 (13)	C9—H9B	0.9800
S1—C2	1.786 (15)	C9—H9C	0.9800
			
Br1—Pb1—Br1^iii^	161.96 (5)	O4—S4—C8	105.2 (4)
Br1^i^—Pb1—Br1^ii^	161.96 (5)	C7—S4—C8	96.1 (4)
Br1—Pb1—Br1^i^	88.591 (8)	O3—S3—Yb1	32.3 (2)
Br1^iii^—Pb1—Br1^ii^	88.591 (8)	O3—S3—C5	104.7 (5)
Br1—Pb1—Br1^ii^	88.591 (8)	O3—S3—C6	105.8 (6)
Br1^iii^—Pb1—Br1^i^	88.591 (8)	C5—S3—Yb1	125.8 (4)
Br2—Pb1—Br1^ii^	99.02 (3)	C5—S3—C6	97.8 (6)
Br2—Pb1—Br1	99.02 (3)	C6—S3—Yb1	120.0 (5)
Br2—Pb1—Br1^iii^	99.02 (3)	O2—S2—Yb1	34.6 (2)
Br2—Pb1—Br1^i^	99.02 (3)	O2—S2—C4	104.8 (5)
S3^iv^—Yb1—S3	125.90 (7)	O2—S2—C3	106.2 (5)
S2—Yb1—S3^iv^	81.32 (5)	C4—S2—Yb1	112.8 (4)
S2^iv^—Yb1—S3^iv^	75.66 (5)	C3—S2—Yb1	134.0 (4)
S2—Yb1—S3	75.66 (5)	C3—S2—C4	97.5 (5)
S2^iv^—Yb1—S3	81.32 (5)	O1—S1—C1	106.0 (6)
S2—Yb1—S2^iv^	128.01 (8)	O1—S1—C2	101.9 (6)
O3^iv^—Yb1—S3	112.72 (15)	C1—S1—C2	96.7 (8)
O3—Yb1—S3^iv^	112.72 (15)	S3—O3—Yb1	126.9 (3)
O3^iv^—Yb1—S3^iv^	20.75 (15)	S1—O1—Yb1	133.0 (4)
O3—Yb1—S3	20.74 (15)	S4—O4—Yb1	134.8 (4)
O3^iv^—Yb1—S2	92.18 (16)	S2—O2—Yb1	123.8 (3)
O3—Yb1—S2	55.45 (15)	S4—C7—H7A	109.5
O3^iv^—Yb1—S2^iv^	55.45 (15)	S4—C7—H7B	109.5
O3—Yb1—S2^iv^	92.18 (16)	S4—C7—H7C	109.5
O3^iv^—Yb1—O3	105.8 (3)	H7A—C7—H7B	109.5
O3—Yb1—O4^iv^	146.6 (2)	H7A—C7—H7C	109.5
O3—Yb1—O4	76.3 (2)	H7B—C7—H7C	109.5
O3^iv^—Yb1—O4^iv^	76.3 (2)	S2—C4—H4A	109.5
O3^iv^—Yb1—O4	146.6 (2)	S2—C4—H4B	109.5
O3—Yb1—O2	71.92 (18)	S2—C4—H4C	109.5
O3^iv^—Yb1—O2^iv^	71.92 (18)	H4A—C4—H4B	109.5
O3^iv^—Yb1—O2	73.06 (19)	H4A—C4—H4C	109.5
O3—Yb1—O2^iv^	73.05 (19)	H4B—C4—H4C	109.5
O1—Yb1—S3^iv^	119.98 (17)	S4—C8—H8A	109.5
O1^iv^—Yb1—S3	119.98 (17)	S4—C8—H8B	109.5
O1—Yb1—S3	91.31 (17)	S4—C8—H8C	109.5
O1^iv^—Yb1—S3^iv^	91.31 (17)	H8A—C8—H8B	109.5
O1—Yb1—S2^iv^	163.81 (17)	H8A—C8—H8C	109.5
O1^iv^—Yb1—S2^iv^	62.82 (16)	H8B—C8—H8C	109.5
O1—Yb1—S2	62.82 (16)	S3—C5—H5A	109.5
O1^iv^—Yb1—S2	163.81 (17)	S3—C5—H5B	109.5
O1—Yb1—O3^iv^	140.5 (2)	S3—C5—H5C	109.5
O1^iv^—Yb1—O3^iv^	85.5 (2)	H5A—C5—H5B	109.5
O1—Yb1—O3	85.5 (2)	H5A—C5—H5C	109.5
O1^iv^—Yb1—O3	140.5 (2)	H5B—C5—H5C	109.5
O1^iv^—Yb1—O1	109.9 (3)	S3—C6—H6A	109.5
O1^iv^—Yb1—O4	74.4 (2)	S3—C6—H6B	109.5
O1^iv^—Yb1—O4^iv^	72.5 (2)	S3—C6—H6C	109.5
O1—Yb1—O4	72.5 (2)	H6A—C6—H6B	109.5
O1—Yb1—O4^iv^	74.4 (2)	H6A—C6—H6C	109.5
O1^iv^—Yb1—O2^iv^	75.0 (2)	H6B—C6—H6C	109.5
O1^iv^—Yb1—O2	146.2 (2)	S2—C3—H3A	109.5
O1—Yb1—O2	75.0 (2)	S2—C3—H3B	109.5
O1—Yb1—O2^iv^	146.2 (2)	S2—C3—H3C	109.5
O4^iv^—Yb1—S3^iv^	59.21 (14)	H3A—C3—H3B	109.5
O4^iv^—Yb1—S3	164.14 (15)	H3A—C3—H3C	109.5
O4—Yb1—S3	59.21 (14)	H3B—C3—H3C	109.5
O4—Yb1—S3^iv^	164.14 (15)	S1—C1—H1A	109.5
O4^iv^—Yb1—S2	91.35 (15)	S1—C1—H1B	109.5
O4^iv^—Yb1—S2^iv^	114.21 (15)	S1—C1—H1C	109.5
O4—Yb1—S2^iv^	91.35 (15)	H1A—C1—H1B	109.5
O4—Yb1—S2	114.22 (15)	H1A—C1—H1C	109.5
O4^iv^—Yb1—O4	120.7 (3)	H1B—C1—H1C	109.5
O4^iv^—Yb1—O2^iv^	135.7 (2)	S1—C2—H2A	109.5
O4—Yb1—O2	135.7 (2)	S1—C2—H2B	109.5
O4^iv^—Yb1—O2	77.2 (2)	S1—C2—H2C	109.5
O4—Yb1—O2^iv^	77.2 (2)	H2A—C2—H2B	109.5
O2^iv^—Yb1—S3^iv^	92.66 (14)	H2A—C2—H2C	109.5
O2—Yb1—S3^iv^	60.01 (15)	H2B—C2—H2C	109.5
O2—Yb1—S3	92.66 (14)	O5—S5—C9	104.8 (10)
O2^iv^—Yb1—S3	60.00 (15)	S5—C9—H9A	109.5
O2—Yb1—S2^iv^	119.48 (15)	S5—C9—H9B	109.5
O2^iv^—Yb1—S2^iv^	21.68 (15)	S5—C9—H9C	109.5
O2^iv^—Yb1—S2	119.48 (15)	H9A—C9—H9B	109.5
O2—Yb1—S2	21.68 (15)	H9A—C9—H9C	109.5
O2—Yb1—O2^iv^	120.2 (3)	H9B—C9—H9C	109.5
O4—S4—C7	105.1 (4)		
			
C7—S4—O4—Yb1	−151.3 (5)	C6—S3—O3—Yb1	−121.5 (5)
C4—S2—O2—Yb1	−108.9 (5)	C3—S2—O2—Yb1	148.6 (5)
C8—S4—O4—Yb1	107.9 (5)	C1—S1—O1—Yb1	105.7 (6)
C5—S3—O3—Yb1	135.8 (5)	C2—S1—O1—Yb1	−153.7 (7)

Symmetry codes: (i) *y*, −*x*+1/2, *z*; (ii) −*y*+1/2, *x*, *z*; (iii) −*x*+1/2, −*y*+1/2, *z*; (iv) −*x*+3/2, −*y*+1/2, *z*.
